# Reflective environment heightens crayfish aggressive and fearful behaviors

**DOI:** 10.17912/micropub.biology.001184

**Published:** 2024-05-24

**Authors:** Stephanie M Rocca, Danielle N Saldana, Merve Addemir, Julianna A Koenig, Bin Z He, Olga L Miakotina, Daniel F Eberl

**Affiliations:** 1 Biology, University of Iowa, Iowa City, Iowa, United States

## Abstract

Animals typically respond to their reflection as a conspecific and will respond as if the reflection were another animal that they could interact with, either fearfully or aggressively. We investigated how a modified reflective environment of a standard glass aquarium affects the aggressive and fearful behaviors of the crayfish *Orconectes virilis* , based on pre-determined behavior criteria. We found that the crayfish were both increasingly aggressive and slightly fearful in the reflective environment compared to minimal behavioral changes in the control non-reflective environment. Thus, our findings support that crayfish recognize their mirror image as a conspecific.

**Figure 1. Crayfish respond to their reflections as conspecifics:**
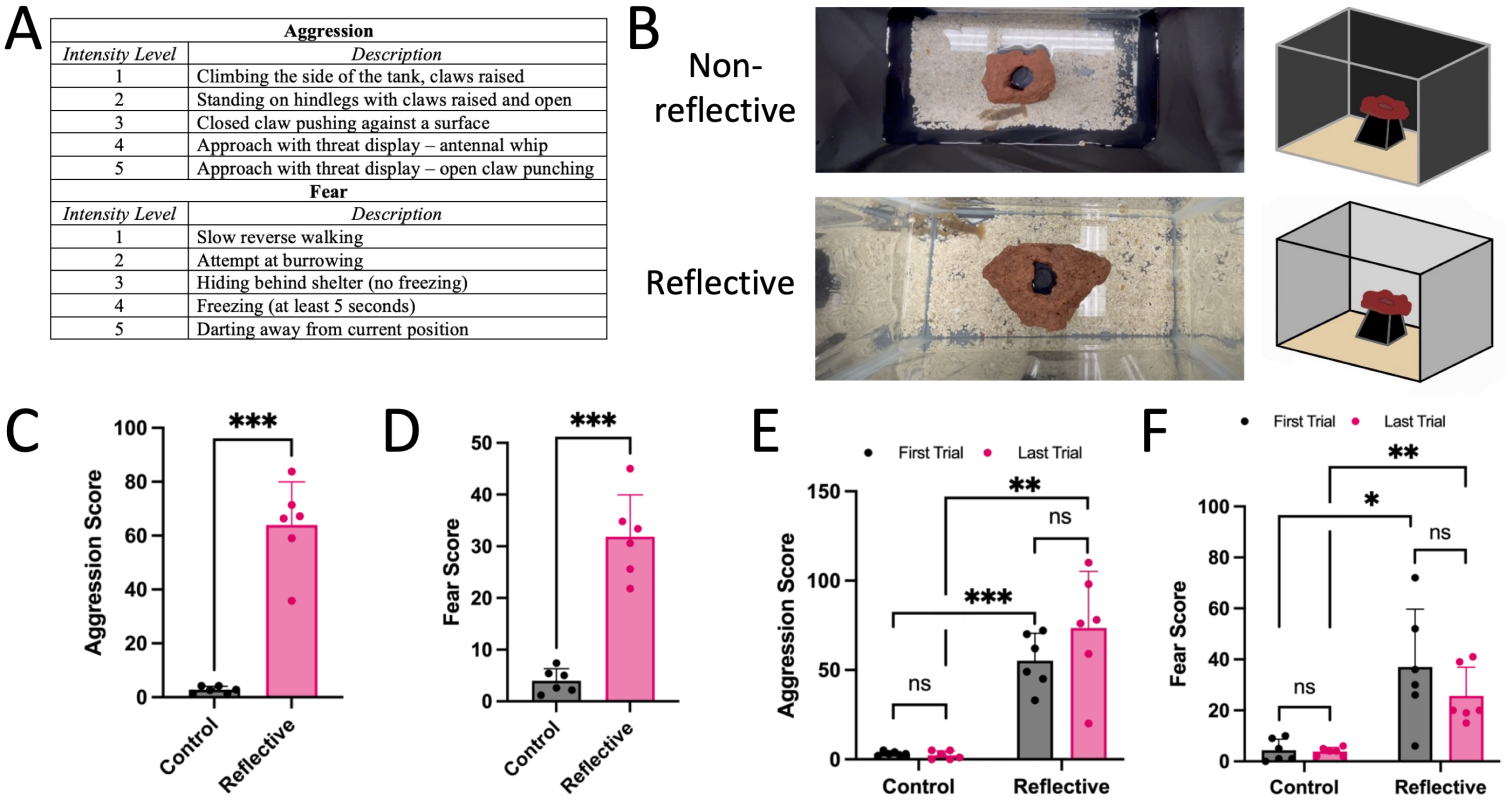
(A) Table of intensity level scores assigned to each behavior observed. Scores are organized in increasing intensities from 1-5 for both aggression and fear. (B) Overhead images of control (upper) and reflective (lower) tank set-ups. (C-D) Comparison of crayfish behavioral scores between conditions. Dots represent mean aggressive (C) and fearful (D) scores of individual crayfish over 5 trials. Bars represent mean + SD of the 6 crayfish (n = 6 crayfish, paired t-test, *** p-value = 0.0002 (C) and 0.0005 (D)). (E-F) Comparison of crayfish summed behavioral scores between first and last trial in both control and reflective environments. Dots represent summed aggressive (E) and fearful (F) scores of individual crayfish. Bars represent mean + SD of the 6 crayfish (n = 6 crayfish, multiple paired t-tests, p-value = 0.036 for control and 0.090 for reflective (E) and 0.793 for control and 0.137 for reflective (F)).

## Description

An organism reacting to its mirror image could have varied responses. These responses suggest that animals can recognize their mirror image as a conspecific. Animals typically act as if the mirror image was another animal that may be a possible peer or threat. For example, through open-field testing, adult mice recognized their reflections as companions and felt less anxiety in a mirrored environment than the nonreflective environment (Zhou et al., 2022) . In addition, the giant panda was unable to recognize their self-image and rather responded aggressively to the threat, which was shown through varied forms of mirror tests (Ma et al., 2015) . Crayfish are a common model organism for aggression studies. Bovbjerg (1953) tested crayfish in the laboratory setting to see how the social hierarchies are established through aggressive behaviors. After support for a dominance order was discovered, a follow-up experiment found that a dominance hierarchy was formed between same size and sex crayfish with size directly relating to dominance in both sexes (Bovbjerg, 1956) . Crayfish have shown that they recognize their mirror image as a conspecific and respond aggressively. In the experiment by Mercier and May, they specifically investigated how socialization vs isolation impacted the crayfish response to their reflections in a singular tank with both reflective and non-reflective regions (May & Mercier, 2007) . It was concluded that socialized crayfish spent more time on the reflective side than isolated crayfish and were more likely to respond to the reflective cues with an approach behavior set. A later experiment by Mercier and May (2010) followed up with these results and found that dominant crayfish spent more time in a reflective environment, suggesting that crayfish recognize the reflection as a conspecific. In contrast, here we focused on the type of responses, both aggressive and fearful.

We asked whether modifying the environment of a standard glass aquarium to be reflective affects both aggressive and fearful behaviors of the crayfish, *Orconectes virilis.* While no previous studies examined agonistic behavior in this species, Tierney et al. (2000) compared four other crayfish species and reported variation in levels of aggressive behavior. We wanted to gain further insight into crayfish responsiveness and familiarization when repeatedly confronted with a reflective environment. We addressed these gaps in knowledge by comparing the behaviors of individual crayfish both in standard and reflective environments, using a scoring system for specific behaviors that we interpreted as aggressive or fearful ( Figure 1A ). Five trials were run with six crayfish individually in two separate tanks where the control tank was lined with black matte non-reflective material and the experimental tank with a reflective material. We predicted a change in crayfish behavior when introduced to the reflective environment where aggression and fear would be increased, which would be observed through expected responses to threatening situations seen in natural habitat.

To investigate the impact of the reflective mirror on crayfish aggressive behavior, the two conditions were compared using averages of the five trials for all crayfish aggression ( Figure 1C ). Behavioral scores in the control tank were neutral, showing overall a lack of aggression. In contrast, in the tank with the reflective mirror, crayfish demonstrated significantly higher aggressive behavioral scores (paired t-test, p = 0.0002). Of the aggressive behaviors, ‘closed claw pushing against a surface’ was the most frequently seen. To investigate the impact of the reflective mirror on crayfish fearful behavior, the two conditions were compared using averages of the five trials for all crayfish fearful actions ( Figure 1D ). Behavioral scores in the control tank were close to neutral, showing overall a lack of fearful actions. Crayfish demonstrated significantly more fearful behavioral scores when placed in the reflective tank compared to the control tank (paired t-test, p = 0.0005). Of the fearful behaviors, ‘slow reverse walking’ and ‘hiding behind shelter’ were the most frequently seen.

Use of the same crayfish individuals in repeated trials raises a concern of potential pseudo-replication. To mitigate this potential, each repeat trial was conducted at least two days after the previous one. Furthermore, statistical analysis found that each trial on its own showed significant effects of the reflective environment on individual trials. For example, in the first and last trials ( Figure 1E and F ), the crayfish demonstrated significantly higher aggressive scores in the reflective tank ( Figure 1E, paired t -tests; p = 0.0003 and p = 0.0024, respectively). Similarly, the crayfish demonstrated significantly higher fearful scores for both trials ( Figure 1F, paired t -tests; p = 0.013 and p = 0.003, respectively). Thus, even without replication over five trials, our conclusions of the crayfish exhibiting heightened behavior in response to the reflective environment are supported.

To investigate the possibility that crayfish responses are enhanced or suppressed over repeated trials, we compared behavioral scores between the first and last trials separately for aggression and fear ( Figure 1E and F ). There was a slight increase in aggressive behaviors shown when comparing the first trial to the last trial of the reflective conditions, whereas there was a slight decrease in fearful behaviors, but these differences were not statistically significant (paired t-tests, p = 0.090 and p = 0.137, respectively). The control behavioral scores remained equally neutral between the first and last trials for both sets of responses (paired t-tests, p = 0.363 and p = 0.792, respectively). Thus, we found no evidence that crayfish significantly changed their responses over the five trials.

While the scoring system developed was used to determine which behaviors were classified as aggressive or fearful, the scores assigned are admittedly subjective. The scoring system was based both on behaviors seen in our experiments and on an ethogram from previous literature (Reisinger et al., 2015) . In that study, it was suggested that an infection with a parasite altered crayfish agonistic interactions which were described by the ethogram. Overall, the crayfish in our study showed more aggressive behaviors than fearful behaviors in the reflective tank. This is supported by the greater number of times aggressive behaviors occurred compared to fearful behaviors (538 aggressive behaviors vs 306 fearful behaviors total over the five trials). More aggression may have occurred due to the crayfish reflection being the same size and sex, as previously reported by Bovbjerg (1956). Thus, the crayfish were more likely to respond as dominants towards the reflection. As per Mercier and May (2010), the hierarchy was established where dominant and social crayfish responded to the reflection as a conspecific whereas the subordinate and isolated crayfish did not interact to similar cues.

To determine whether, even if each behavior were unweighted, the crayfish would still show significantly different behavior in the reflective environment compared to the non-reflective environment, we ran a paired t-test on behavior counts. Using the averaged counts of behaviors across the five trials compared between the control and reflective environments, an aggressive p-value of 0.0002 and a fearful p-value of 0.0007 were calculated, confirming a significant difference for both. Thus, our conclusion, that the crayfish were significantly more aggressive and fearful in the reflective environment, remains supported. The results shown in all figures support our hypothesis that crayfish behavior would change when shifted from the control to the reflective environment. Our results show an increased magnitude of both aggressive and fearful behaviors. This suggests that the crayfish recognize their reflection as a conspecific rather than as themselves. These results are consistent with the experiment of Drozdz et al. (2006) where crayfish housed in pairs showed significantly more behavioral responses and time in front of mirrors than in non-mirrored portions of the tank. Our results also suggest that crayfish lack the ability to recognize their own reflection as self under our experimental conditions.

It is important to acknowledge that there were cases of distractions from movement in front of the open side of each tank, which could have contributed to differences seen in crayfish behavior as a response. Also, there was a notable increase of stress when handling the crayfish between trials. Thus, the behavioral scores could have been impacted to be more aggressive or fearful, rather than due to the reflective vs non-reflective environment. These are demonstrations of how animal behavior is often unpredictable and could be heavily influenced by human intervention. To decrease stress, crayfish could be held in one tank during an active trial, introducing the reflective material after crayfish are acclimated to avoid transferring them between trials.

A future experiment worth investigating is crayfish capability associated with memory. More trials could be run with varying time lengths in between to see how long it takes for the crayfish to habituate to their reflections through exhibiting subdued behaviors. Though the behaviors we see are not those expected from self-recognition, we cannot rule it out from our results. A mark test could be applied as was done in the ghost crab (Robinson, 2023) . Regarding social influence, past studies have suggested that social crayfish tend to be more reactive than isolated crayfish to the mirrored side of the tank (Mercier & May, 2010) . Future studies could test how grouped crayfish placed into a mirrored environment would react to the reflections surrounding them vs the singular crayfish trials run in this experiment.

In conclusion, the aggression and fear that crayfish exhibited in this experiment was a result of insufficient recognition of their mirrored image as themselves but rather as a conspecific.

## Methods

Crayfish from the species *Orconectes virilis* were sourced from Carolina Biological Supply. Three male and three female crayfish, approximately 3 inches in length, were used. All crayfish were housed in a 32 Qt (L) flat plastic container, with dividers to avoid fighting between the crayfish, and plant pot shelters for hiding. The dividers were perforated to facilitate oxygen and water circulation. Water was filled to a four-inch mark on the tank with an aerator in every other chamber, totaling to three. The crayfish were each fed 3 pellets of New Life Spectrum Large Fish Formula every other day.

The experimental trials were conducted using two 5-gallon glass aquarium tanks (L 16 in x H 10 in x D 8 in). The control tank had three sides covered in a matte black, non-reflective material. The experimental tank had three sides covered in a reflective material. One side was left uncovered on each tank to allow for further observation. Both tanks had a thin layer of gravel to avoid external unintended stress on the animal. In addition, an obstacle consisting of an overturned square plastic plant pot, held in place with a rock, such that the crayfish could avoid direct view of the reflective or non-reflective surfaces while staying in view of the camera (see Figure 1B for overhead images ). When running trials, each crayfish was placed into the control tank for data recording of 10 minutes. After the recording, the crayfish was placed back into the housing environment to reacclimate to a relaxed state for an estimated 50-60 minutes. After all six crayfish were tested in the control tank, they were then tested in the same order in the reflective tank for 10 minutes. To minimize stress, each crayfish experienced trials that occurred every other day over 1.5 weeks, summing to five total sets of trials.

For both conditions, a mobile phone attached to a stand was used to record the behaviors from above. Video footage was viewed and replayed to ensure all behaviors were accounted for. Based on the video, we manually counted all the behaviors and assigned an aggression score for each observation based on Figure 1A . We then summed the total score per animal per trial, which we termed the summed aggression score. In Figure 1C and D, we averaged the summed score for each animal over the five trials such that for each animal, we have a mean summed score under each of the control and reflective conditions . Using GraphPad Prism, paired t-tests were conducted and two-tailed *p* -values are reported. For Figure 1E and F, we used the summed scores for only the first trial and the last trial for each animal . The same statistical testing procedure was applied. All data passed normality tests, with the exception of the reflective environment fear scores in the last trial, in which the normality test resulted in a marginally significant p-value = 0.043.

## Extended Data

Description: Video showing selected examples of crayfish behaviors in control and reflective environments.. Resource Type: Audiovisual. DOI: 10.22002/w5d1d-py956
